# Sex- and gender-related differences in systemic lupus erythematosus: a scoping review

**DOI:** 10.1007/s00296-025-05910-7

**Published:** 2025-06-27

**Authors:** Katinka Albrecht, Wiebke Troll, Johanna Callhoff, Anja Strangfeld, Sarah Ohrndorf, Johanna Mucke

**Affiliations:** 1 German Rheumatology Research Center Berlin, Program Area Epidemiology and Health Services Research, Berlin, Germany; 2 Augenärztliches MVZ Fetscherplatz/Lockwitzgrund Dresden, Dresden, Germany; 3 Department of Rheumatology and Clinical Immunology, Charité Universitätsmedizin Berlin, Berlin, Germany; 4 Department of Gastroenterology, Hepatology, Infectious Diseases and Rheumatology, Ernst von Bergman Hospital Potsdam, Potsdam, Germany; 5 HMU Health and Medical University Potsdam, Potsdam, Germany; 6 Department of Rheumatology, University Hospital Düsseldorf, Medical Faculty of Heinrich Heine University, Düsseldorf, Germany; 7 Ruhr Universität Bochum, Rheumazentrum Ruhrgebiet, Herne, Germany

**Keywords:** Antirheumatic agents, Gender differences, Patient-reported outcomes, Sex differences, Systemic lupus erythematosus

## Abstract

A scoping review was conducted to compile evidence on sex-specific differences in systemic lupus erythematosus (SLE) with focus on autoantibodies, organ manifestation, damage, treatment and patient-reported outcomes (PROs). Systematic searches in PubMed and Cochrane were performed including meta-analyses, observational studies and clinical trials from 01/2015 to 11/2024. Studies of adults with SLE reporting outcomes by sex were eligible. The research protocol is registered in the Registry for Scoping Reviews (OSF, https://osf.io/gfbs9). From 373 screened articles, 81 publications were included. Studies comprised differences in autoantibodies (*n* = 13), damage (*n* = 40), organ involvement (*n* = 27), treatment (*n* = 14), and PROs (*n* = 6). Twenty studies compared proportions of outcomes by sex with patient numbers ranging from 98 to 11,943. The female/male ratio was between 4:1 and 11:1. The review found a higher age at onset in men and a higher proportion of positive lupus anticoagulant, nephritis, serositis, antiphospholipid syndrome, greater renal and cardiovascular damage and severe infections. SLE in women more often presented with Ro/SSA autoantibodies, alopecia, photosensitivity, Raynaud, and osteoporosis. Some studies showed more frequent cyclophosphamide and less frequent antimalarials in men. Little evidence indicated more frequent non-adherence with azathioprine and mycophenolate in women. Limited evidence was available for PROs. This review confirms significant sex differences in SLE, with men showing later onset, more severe organ damage, and distinct autoantibody and treatment patterns, while women more often present with Ro/SSA autoantibodies, photosensitivity, and osteoporosis. Evidence on patient-reported outcomes remains limited, highlighting the need for further research to guide sex-specific management.

**Supplementary Information:**

The online version contains supplementary material available at 10.1007/s00296-025-05910-7.

## Introduction

Systemic lupus erythematosus (SLE) is an autoimmune inflammatory rheumatic disease affecting mostly women. In all nationalities females predominate with a ratio ranging between 1.2:1 and 15:1 [1]. Small numbers for men limit the investigation of sex-related differences in clinical trials but a growing number of cohort studies show different manifestations in women and men.

While most studies indicate a tendency for men to experience more severe disease manifestations and greater organ damage in SLE, some research suggests otherwise. Systematic reviews have been conducted to examine sex differences, primarily focusing on clinical manifestations [2–4]. However, beyond these well-studied aspects, there is growing evidence that sex differences also extend to the antibody profile, comorbidities, rarer disease manifestations, and patient-reported outcomes (PROs). Despite their significant implications for disease management, these aspects have not yet been comprehensively summarized.

The objective of this scoping review is to provide a broader perspective on sex differences in SLE by mapping existing research across multiple dimensions. Specifically, we aim to synthesize evidence on differences in autoantibodies, organ manifestations, damage, PROs, and treatment approaches.

## Methods

A systematic literature research was performed in PubMed and Cochrane. English peer-reviewed publications from 01/2015 to 11/2024 were considered. Eligibility criteria included adults with SLE, systematic reviews, observational studies and clinical trials with ≥ 20 men, comparing age at onset, autoantibodies, organ manifestation, damage, PROs, treatment adherence, response, efficacy and safety (Supplementary table S1). If results were reported in different publications from the same cohort, the more recent study was selected.

### Search strategy

The search strategy was developed in several steps to capture available evidence without including an excessive number of non-applicable studies. The ubiquitous occurrence of the terms female and sex in almost every SLE study led us to not search for these terms in the main text. Through repeated testing of different search terms, we finally selected a search strategy with an acceptable number of hits (Supplementary table S2). Two reviewers (KA, JM) independently screened titles, abstracts and full-texts according to the pre-defined criteria. Disagreements were resolved by discussion. Additional records were identified through supplementary searches on references and conference abstracts.

### Data extraction

Study characteristics were extracted by study design, origin, period, number of women and men and ethnicity (Supplementary table S1).

### Statistics

We extracted outcomes as proportions, means, or median values. Odds ratios (OR) were calculated as the percentage of men divided by the percentage of women with the corresponding item being present. To show clinically relevant differences, results with OR ≥ 1.4 were considered more common in men and OR ≤ 0.7 more common in women. For mean values, minimal clinically important differences (MCID) were used, considering > 3 points difference for Systemic Lupus Erythematosus Disease Activity Index (SLEDAI) [5], > 0.3 for Systemic Lupus International Collaborating Clinics/American College of Rheumatology (SLICC/ACR) Damage Index (SDI) [6] and > 2 for European Consensus Lupus Activity Measurement (ECLAM) [7] and Physician Global Assessment (PGA) [7] between women and men as relevant. We do not report significance in form of p-values as a criterion because these not necessarily reflect clinically meaningful differences [8]. Adjusted values were considered, e.g. incidence ratios (IR), OR, hazard ratios (HR) or relative rates (RR).

Extracted data was summarised in narrative and table syntheses. We provide a colored illustration of differences, adapted from Coates et al. [9], to present the results of a large number of very different studies in a concise way. Since we only extracted descriptive frequency data and did not use them in the form of a meta-analysis, we did not perform a critical appraisal of the included studies as long as the inclusion criteria were met. The review was conducted following the updated methodological guidance for the conduct of scoping reviews [10] and the PRISMA Extension for Scoping Reviews (PRISMA-ScR) [11]. The research protocol was registered in the Registry for Scoping Reviews (OSF, https://osf.io/gfbs9). A patient partner (WT) was involved in all stages of the study.

## Results

From 373 identified records, 81 full-text articles were included (see flow chart in Supplementary figure S1). Differences regarding organ manifestation and damage were most frequently reported. Few studies reported differences in PROs and treatment.

### Study characteristics

Six meta-analyses, four systematic reviews, 62 observational studies, one post-hoc analysis and three case-control studies were included. Of these, 20 studies compared proportions of outcomes by sex. In these studies, patient numbers ranged from 98 to 11,943, with a female/male ratio between 4:1 and 11:1 (Supplementary table S3). Study origin and ethnicity varied across studies. Nine studies reported the mean age at onset in men (range 25 ± 11 years to 44 ± 21 years) and women (26 ± 10 to 36 ± 15 years). In seven of nine studies, men had a higher age at disease onset between 2 and 11 years. In cohorts comparing early versus late onset, men had a higher proportion of late onset [12]. In three cohorts, time to diagnosis was longer in females [13–15], in one cohort it was equal [16] and in another cohort it was longer in men [17]. The other included studies reported sex as a risk factor for a specific outcome or other sex-related differences. They are included in the narrative synthesis.

### Autoantibodies

One meta-analysis and 12 cohort studies compared the presence of autoantibodies in women and men (Table 1). In 5 of 6 studies, Lupus Anticoagulant (LA) occurred with higher odds in men than women (OR 1.3 to 2.0). Anti-Cardiolipin antibodies were slightly more often present in men (OR between 1.1 and 1.3 and 1.7 in one study). In 6 of 9 studies, anti-Ro/SSA autoantibodies occurred more often in women. The presence of other autoantibodies was not consistently different between the two sexes (proportions see Supplementary table S4).

**Table 1:**
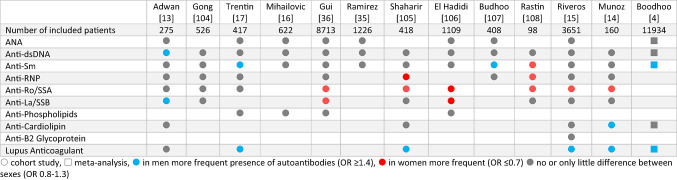
Sex differences in autoantibodies

### Disease activity

Levels of disease activity markers were compared in seven cohort studies and one meta-analysis (Table 2). Mean or median SLEDAI values were equal or higher in men compared to women. Modeling long-term outcomes from the Hopkins Lupus cohort showed no significant effect of sex on the change in SLEDAI over time [18]. One cohort and a meta-analysis found low C3 values more frequent in men [4, 13]. Mean ECLAM values were compared in one cohort with higher values in men [19] (Supplementary table S5).

**Table 2:**
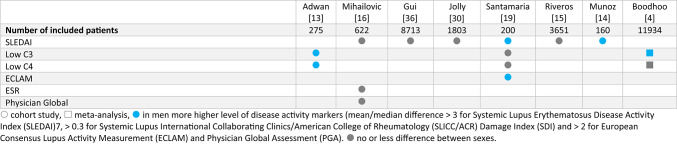
Sex differences in disease activity markers

### Clinical manifestation

Different outcomes regarding clinical manifestations were reported in three meta-analyses and 14 cohort studies (Table 3). In six out of six studies, women had alopecia more often than men and in four of five studies, Raynaud was more frequent in women. Two meta-analyses found a higher proportion of photosensitivity in women. A slightly more frequent occurrence in women was also shown with malar rash, whereas nephritis, serositis, venous thrombosis and APS were more frequent in men (Supplementary table S6). In a retrospective Chinese cohort, thrombocytopenia was more commonly found in young females [20], contrasting the result of the other cohort studies.

**Table 3:**
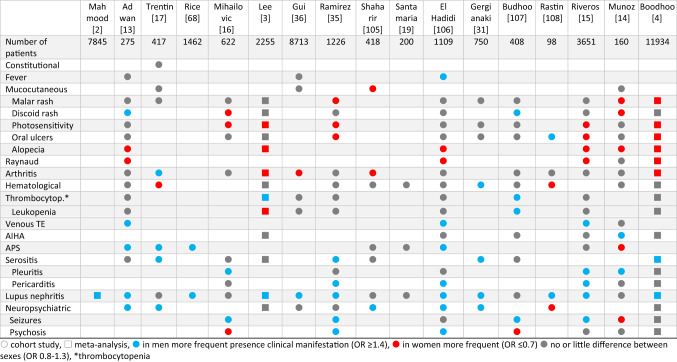
Sex differences in clinical manifestation

### Lupus nephritis (LN)

A recent meta-analysis on sex differences in the outcome of LN included 20 studies reporting kidney histopathology [2]. The data confirmed results from cohort studies that compared to women, men have a greater risk of diffuse proliferative LN class IV/V, worse renal outcomes and a lower chance of complete remission. A meta-analysis on Asian SLE patients confirmed a greater incidence of renal involvement and thrombocytopenia in men compared to women [3]. In a Danish risk calculation study for the development of proteinuria, men with lymphopenia had the highest predicted risk of proteinuria [21]. A prediction model for a 10-year risk of LN included male sex as risk factor [22].

### Serositis

In seven cohort studies and two meta-analyses, serositis was more common in men, but did not reach an OR > 1.4 in all studies. A Spanish case-control study also revealed male sex as risk factor for serositis, but women were at higher risk for recurrent serositis [23]. Male sex was an independent significant risk factor for pleuritis, but not for pericarditis in a Chinese cohort study [24].

### Neuropsychiatric involvement

The Systemic Lupus International Collaborating Clinics (SLICC) cohort revealed male sex as a risk factor for neuropsychiatric events [25, 26]. The distribution in the other cohort studies is inconsistent regarding the occurrence of psychosis, but with a higher occurrence of seizures in men (supplementary table S6).

### Damage

Damage was assessed very differently, in terms of definition, item selection and methodology. In nine cohorts, the proportion of damage was compared in men and women, showing an overall higher proportion of damage in men (Table 4). A multivariate analysis from the RELESSER cohort found male sex as a risk factor for refractory disease [27]. Data from the CSTAR cohort showed that elderly men with a history of major organ involvement at SLE diagnosis were at the highest risk of severe flares and organ damage [28]. An SLR on the SLICC damage index (SDI) found male sex associated with higher SDI scores, also in studies that adjusted for age and disease duration [29]. In four more recent cohorts, men had higher SDI values [16, 30–32], the other studies showed no relevant difference [17, 33–36] (Supplementary table S7).

**Table 4:**
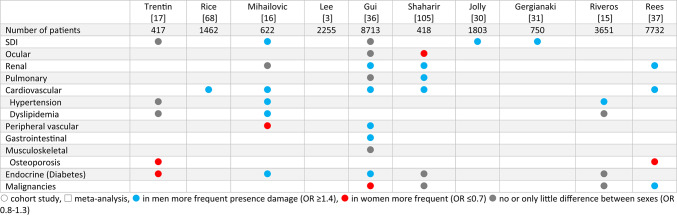
Sex differences in organ damage

### End stage renal disease (ESRD)

The UK CPRD database and a cohort from Taiwan showed a higher incidence rate of ESRD in men compared to women with SLE. The relative risk compared to the general population was also higher in men [37, 38]. Hypertension was identified as a more significant risk factor for ESRD in women in the Taiwanese cohort. A meta-analysis including 11 studies on nephritis progression found that in women increased serum uric acid levels were associated with higher risk of LN progression [39]. No differences in ERSD between men and women were observed in patients with incident LN using a large US claims database [40].

### Cardiovascular (CV) disease

Male sex was confirmed as risk factor for CV disease in SLE in an umbrella review comprising the results from three systematic reviews [41]. A meta-analysis from 2015 including 32 studies identified male sex as a predictor of CV events in SLE (OR 6.2 [1.49-25]) [42]. A SLR for coronary heart disease also revealed male sex as risk factor in SLE [43]. All recent cohort studies consistently confirm a higher risk of CV damage in men compared to women. Population-based US registry data found the highest rates of CV events in men younger than 50 [44]. Male sex was specifically found as independent risk factor for atrial fibrillation, valvular heart disease, aortic aneurysm/dissection and thrombotic events [45–48]. Data from a US cohort with hospitalized SLE patients identified that young individuals aged 20–29 and among them particularly women had the greatest odds of atherosclerotic CV disease compared to sex and age-matched controls [49]. A risk prediction model from the CSTAR cohort included male sex as risk factor for CV and cerebral events [50].

### Pulmonary

Male sex was associated with having pulmonary disease identified on chest X-ray in a retrospective cohort [51].

### Autoimmune hemolytic anemia (AIHA)

In a Latin America cohort male sex was a risk factor for severe AIHA (HR 2.26 [1.02–4.75]) [52].

### Infections

The UK CPRD database showed a higher incidence rate of infections in women with SLE compared to men [37]. Within Taiwanese electronic health record-data, male sex was identified as a risk factor for Pneumocystis jirovecii pneumonia (PJP) infection in SLE (HR 2.42 [1.31;4.46]) [53]. Male sex was a strong risk factor for COVID-19 [54] and COVID-outcomes were found to be more severe in male SLE patients [55, 56]. Men had a higher risk of tuberculosis in an Indian SLE cohort [57]. In a Chinese SLE cohort, latent tuberculosis infection was numerically more frequent in men [58].

### Osseous damage

The UK CPRD database showed a higher absolute risk of osteoporosis in women with SLE compared to men, but the relative risk compared to the general population was higher for men [37]. Within Korean claims data, a threefold increased risk of osteoporotic fractures was found in SLE patients compared to non-SLE controls. Male patients had a relatively higher fracture risk [59]. In a US-electronic health records analysis, bone mineral density (BMD) was less likely tested in male SLE patients [60]. A meta-analysis showed that both female and male SLE patients had a lower BMD at any region (lumbar spine, total hip and femoral neck) than healthy controls [61]. Taiwanese claims data showed an increased risk of osteomyelitis in men compared to women with SLE [62]. In the longitudinal US Hopkins Lupus cohort, men with SLE had an increased risk for osteonecrosis compared to women [63].

### Malignancies

Within the SLICC cohort and the UK CPRD database, overall cancer risk was related to male sex [37, 64]. Analyses of Korean claims data and of data from the Spanish RELESSER cohort identified a higher lymphoma risk in men compared to women with SLE [65, 66]. Regarding hematologic malignancies, the prognosis for men with SLE was poorer in a Chinese case-control study [67].

### PROs

 The review revealed six publications on PROs (Table 5). Within a large US cohort, men more often lacked confidence in completing medical forms and more frequently required help in reading health-related materials. Moderate to severe pain and fatigue was more frequent in women [68]. Differences regarding Quality of Life were assessed within the worldwide Study of Outcomes in Lupus (SOUL) cohort. While men performed worse in the social support domain, women, particularly those in the reproductive age group, assessed worse in cognition and procreation domains with trends for worse functioning on physical health and pain-vitality domains [30]. Sex differences in anxiety and depressive symptoms were studied by Macedo et al. They found worse mental health and vitality with more common depressive symptoms and anxiety in women [69–71]. Workability was examined in a meta-analysis [72]. Only two studies reported sex differences on workability: in a survey from the initial 2000ers, women had a higher risk of work loss [73]. In a US cohort study from 2007, men more frequently reported inability to work [74] (Supplementary table S8).

**Table 5:**
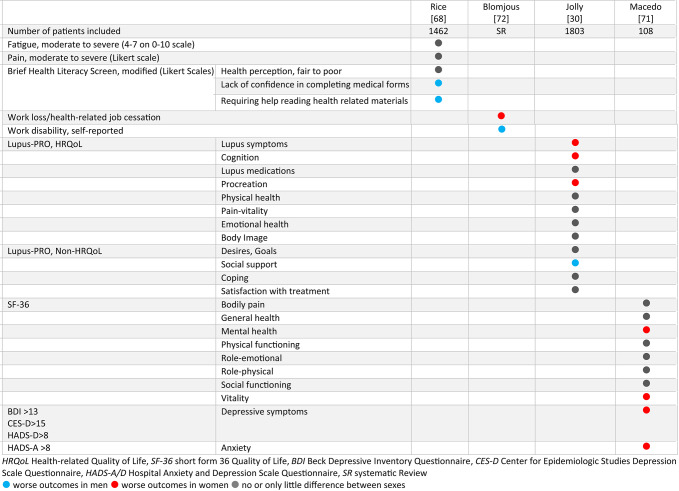
Sex differences in patient-reported outcomes

### Treatment

Seven cohort studies reported data on treatment choices in women and men (Table 6). Cyclophosphamide was applied more frequently in men [15, 19, 36]. Three cohorts reported a less frequent use of antimalarials in men [19, 75, 76] and male sex was a negative predictor of filled hydroxychloroquine prescriptions in US Medicare data [77]. The frequency of GC use and dosing, either at treatment initiation or over the disease course, reported in seven cohorts, was comparable between women and men [15–17, 19, 36, 76]. A modeling from the Hopkins cohort showed no significant effect of sex on the average annual GC dose over time [18].

**Table 6:**
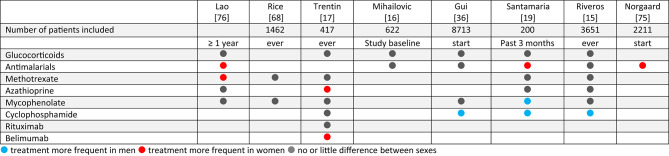
Differences in proportion of treatments for women and men with SLE

Adherence to therapy was examined with US claims data, showing higher non-adherence with azathioprine and mycophenolate in women [78], but no sex difference in adherence with antimalarials [79]. The latter was confirmed in the New Zealand cohort study [76].

Regarding safety, the infection risk under belimumab, rituximab or non-biologic immunosuppressives was numerically higher for men in the British BILAG-register in an unadjusted model [80]. In post hoc data from five belimumab trials, men had a higher risk for neuropsychiatric flares (HR 3.26 [1.51; 7.04]) [81]. No study compared treatment response in women and men.

### Transgender

A scoping review summarised data from seven case reports referring to transgender individuals with SLE [82]. All incident SLE cases occurred in transgender women after estrogen therapy, with variable clinical manifestation. Positive ANA titers are described in up to 30% in transgender populations [83].

A summary of findings is presented in Table 7.

**Table 7:**
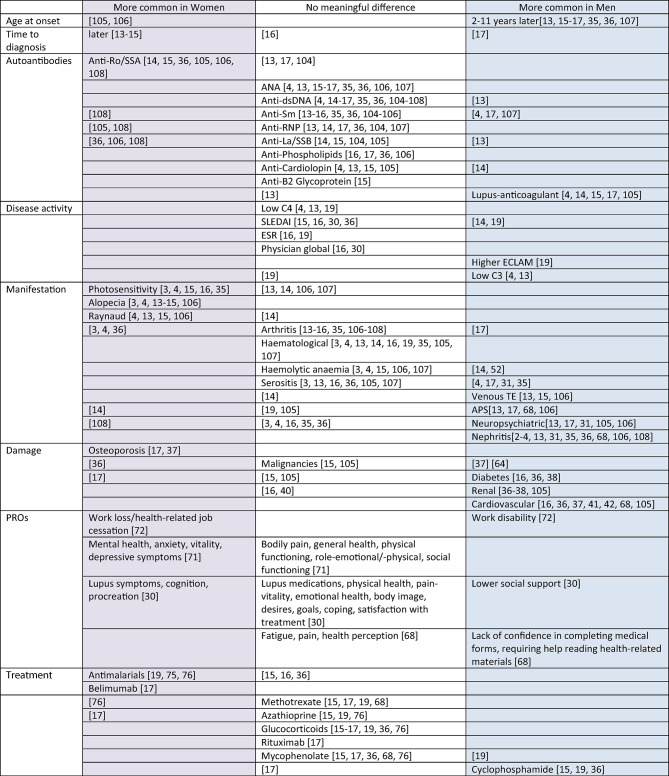
Summary of findings

## Discussion

This review summarizes the evidence on sex- -related differences in SLE. Key findings include a later disease onset in men, a higher occurrence of specific antiphospholipid antibodies, severe organ involvement and greater disease damage in men in contrast to a higher occurrence of positive Anti-Ro/SSA-antibodies, photosensitivity, alopecia and Raynaud in women.

The causes of sex-specific differences in SLE remain complex, involving genetic, hormonal, and epigenetic factors that influence immune system variations between men and women. While these factors explain the female predominance, they do not fully account for differences in disease manifestations. Testosterone has been shown to inhibit dsDNA antibody production and, to a lesser extent, overall antibody production in B cells [84]. However, most studies reviewed found no significant differences in dsDNA antibody positivity, with only one study reporting higher prevalence in men. In contrast, women exhibited higher frequencies of anti-Ro/SSA autoantibodies, consistent with the more common occurrence of secondary Sjoegren’s syndrome [85]. This may be linked to estrogen, which promotes plasma cell proliferation and autoantibody production [86]. Other antibody types occurred at similar rates.

The predominance of LA and secondary APS in men with SLE remains unclear. APS may occur as a primary condition or as secondary APS linked to SLE, with a more pronounced female predominance in secondary (7:1 female-to-male ratio) compared to primary APS (3.5:1) [87]. Notably, men more often exhibit double positivity for LA and anti-cardiolipin antibodies, though single LA positivity is less common [88]. Men also show a higher prevalence of CV diseases, likely related to more frequent smoking [17], which increases arterial event risks in LA-positive individuals and may also contribute to higher APS manifestation rates. LN in men more likely progresses to ESRD, partially linked to comorbidities like hypertension, dyslipidemia, and diabetes, although these do not explain the higher occurrence of LN itself. Men are more prone to serositis and severe organ-threatening manifestations like LN and neuropsychiatric lupus, and accumulate more disease damage overall. Detection bias has been suggested, with delayed diagnosis in men potentially allowing severe complications to develop [35, 89]. However, some studies report shorter diagnosis times in men [13–15], challenging this theory. The increased occurrence of serositis has been linked to a single nucleotide polymorphism in the CXCR3 gene, located on the X chromosome, potentially impacting men more significantly [90].

Severe complications, such as CV events, ESRD, severe infections, lymphoma, and osseous damage, are more common in men and the mechanisms behind CV complications require further research [91]. In women, atypical myocardial infarction symptoms and a higher risk of atherosclerotic CV disease, especially in younger patients [49, 92], highlight the need for tight CV risk management in both sexes. Women also frequently experience osteoporosis, warranting regular bone health assessments for all SLE patients. Men with SLE are more prone to severe infections, including during the COVID-19 pandemic, a trend consistent with the general population. Women’s stronger innate and adaptive immune responses provide better protection against infections but also contribute to increased autoimmunity, while men are generally more vulnerable to severe infections [93].

Sexual dysfunction in men with SLE [94–96] is another important concern, though it was not included in the reviewed literature due to the lack of comparative data.

Treatments show men more frequently receiving cyclophosphamide, used for severe disease, and less frequent use of antimalarials. Data on sex-specific therapeutic responses are lacking, emphasizing the need for subgroup analyses in RCTs to evaluate treatment efficacy for both sexes. Medication adherence, a known challenge in SLE [97], is critical for treatment success. While some evidence suggests lower adherence in women, further research is needed to confirm this finding.

While there is extensive data on clinical differences in SLE, a research gap remains regarding PROs. Initial findings suggest men face greater challenges with social support, while women report higher levels of mental health symptoms. These patterns align with broader gender differences in behavioral, cognitive, and emotional dimensions [98]. Men may also feel discomfort discussing a predominantly female-associated disease, limiting their willingness to seek social support. These findings highlight the need to address both clinical and psychosocial aspects of SLE with gender-sensitive care approaches. Understanding how men and women experience and cope with the disease is crucial for providing holistic care tailored to their specific needs.

Some studies evaluated the validity of diagnostic and quality-of-life tools regarding gender. The EULAR/ACR 2019 criteria performed well in diagnosing SLE across genders [99, 100] similarly to the LupusPro tool, which demonstrated generalizability to both women and men [101].

### Limitations and strengths

This review highlights sex differences in SLE while considering regional and ethnic variations through included studies. While the breadth of examined outcomes may have led to the omission of studies, this likely does not impact the overall findings. The heterogeneity of studies, with a significant female-to-male ratio disparity in SLE poses methodological challenges. Small male sample sizes hinder meaningful sex-based analyses, though large EHR cohorts have improved data availability. This issue is pronounced in RCTs, which rarely stratify outcomes by sex. A recent RCT on mycophenolate acknowledged sex-specific outcomes but lacked sufficient male participants to estimate risks [102]. Men comprise 9% of SLE cases but only 7% in RCTs [103], underscoring the need for more inclusive studies.

### Clinical implications

Future recommendations for SLE should emphasize gender-sensitive care. Increasing awareness of SLE in men is crucial for timely diagnosis, along with intensive treatment for renal, cardiovascular, and neuropsychiatric involvement, proactive management of sexual dysfunction, and tailored social support. For women, attention to atypical myocardial infarction symptoms, mental health concerns, and workplace challenges is essential. Screening for osteoporosis and cancer, with a focus on lymphoma in men, should be prioritized for all patients. Ensuring equitable access to antimalarial therapies is critical. Healthcare providers should consider the potential development of SLE in transgender women undergoing hormone replacement therapy. Addressing both clinical and psychosocial aspects will enable more inclusive and personalised care for all patients.

## Electronic supplementary material

Below is the link to the electronic supplementary material.

Supplementary Material 1

## Data Availability

The extracted data are available in the supplementary tables.
