# Supplementary material for: Sex- and gender-related differences in systemic lupus erythematosus: a scoping review
Source: Rheumatol Int. 2025 Jun 27;45(7):160. doi: 10.1007/s00296-025-05910-7 (PMC12204902; doi:10.1007/s00296-025-05910-7)
Supplement: Supplementary file 1 — Supplementary Material 1 [file 296_2025_5910_MOESM1_ESM.pdf]

Supplementary table S1: Eligibility Criteria

	Inclusion criteria	Exclusion criteria
Population	Adult persons with systemic lupus erythematosus (SLE)	Age < 18 years
Intervention	None	Not applicable
Comparison	Comparison of outcomes by sex or outcomes are reported by sex	Results reported only for females or males
Outcomes	*Presence of autoantibodies*: antinuclear antibodies (ANA), anti-double stranded DNA (anti-dsDNA), anti-Smith (anti-Sm), anti-Ribonucleoprotein (anti-RNP), anti-Sjoegren's-related syndrome antigens (anti-Ro/SSA, anti-La/SSB), anti-Phospholipids (anti-Cardiolipin, anti-Beta2 glycoprotein and Lupus Anticoagulant (LA))<br>Disease activity markers: Systemic Lupus Erythematosus Disease Activity Index (SLEDAI), low complement levels C3 & C4, European Consensus Lupus Activity Measurement (ECLAM), Erythrocyte Sedimentation Rate (ESR), and Physician Global Disease Activity (PGA)<br>*Organ manifestation*: constitutional, fever, mucocutaneous, malar rash, discoid rash, photosensitivity, oral ulcers, alopecia, Raynaud, musculoskeletal, arthritis, hematological, thrombozytopenia, leukopenia, venous TE, hemolytic anemia, antiphospholipid syndrome (APS), serositis, pleuritis, pericarditis, nephritis, neurologic/neuropsychiatric, seizures and psychosis<br>*Damage*: Systemic Lupus International Collaborating Clinics/American College of Rheumatology (SLICC/ACR) Damage Index (SDI), ocular, neuropsychiatric, renal, pulmonary, Cardiovascular (CV), hypertension, dyslipidemia, peripheral vascular, gastrointestinal, musculoskeletal, osteoporosis, skin, endocrine (diabetes), gonadal and malignancies<br>*Patient-reported outcomes*: pain, fatigue, depressive symptoms, anxiety, items on Quality of Life (QoL), workability<br>*Treatment*: glucocorticoids, antimalarials, methotrexate, azathioprine, mycophenolate, ciclosporin A, cyclophosphamide, rituximab, belimumab, anifrolumab	Incidence, prevalence<br>Pregnancy outcomes<br>Operative outcomes<br>cellular, genetic, histologic, hormonal outcomes<br>healthcare access and utilisation, mortality
Study design	Metaanalyses, systematic reviews, observational prospective or retrospective cohort studies, database or registry studies, cross-sectional studies, interventional trials (RCTs, non-RCTs), Studies with a population of ≥ 20 males	Modelling studies, economic evaluations, Case reports, case series, narrative reviews, editorials, commentaries
Language	Full-text articles in English language	
Other	Studies on human subjects<br>Publication date 2015 - 2024	Animal studies

Supplementary table S2: Search Terms

PubMed Search History:
Search number,Query,Sort By,Filters,Search Details,Results,Time
9,#7 NOT #10,Most Recent,"Abstract, English, Humans, from 2015/1/1 - 3000/12/12","(((""sex""[Title] OR ""gender""[Title] OR ""sex""[MeSH Terms] OR (""sex""[MeSH Terms] OR ""gender identity""[MeSH Terms]) OR ""male""[Title/Abstract] OR ""men""[Title/Abstract]) AND (""systemic lupus erythematosus""[Title] OR ""SLE""[Title]) AND (""hasabstract""[All Fields] AND ""humans""[MeSH Terms] AND 2015/01/01:3000/12/12[Date - Publication] AND ""english""[Language])) NOT ((""case reports""[Publication Type] OR ""editorial""[Publication Type]) AND (""hasabstract""[All Fields] AND ""humans""[MeSH Terms] AND 2015/01/01:3000/12/12[Date - Publication] AND ""english""[Language]))) AND ((fha[Filter]) AND (humans[Filter]) AND (2015/1/1:3000/12/12[pdat]) AND (english[Filter]))",364,08:09:58
8,"(""case reports""[Publication Type] OR ""editorial""[Publication Type])",Most Recent,"Abstract, English, Humans, from 2015/1/1 - 3000/12/12","(""case reports""[Publication Type] OR

""editorial""[Publication Type]) AND ((fha[Filter]) AND (humans[Filter]) AND (2015/1/1:3000/12/12[pdat]) AND (english[Filter]))","312,361",08:09:07
7,#1 AND #2,Most Recent,"Abstract, English, Humans, from 2015/1/1 - 3000/12/12","((""sex""[Title] OR ""gender""[Title] OR ""sex""[MeSH Terms] OR (""sex""[MeSH Terms] OR ""gender identity""[MeSH Terms]) OR ""male""[Title/Abstract] OR ""men""[Title/Abstract]) AND (""systemic lupus erythematosus""[Title] OR ""SLE""[Title])) AND ((fha[Filter]) AND (humans[Filter]) AND (2015/1/1:3000/12/12[pdat]) AND (english[Filter]))",434,08:06:06
6,#1 AND #2,Most Recent,"Abstract, English, Humans","((""sex""[Title] OR ""gender""[Title] OR ""sex""[MeSH Terms] OR (""sex""[MeSH Terms] OR ""gender identity""[MeSH Terms]) OR ""male""[Title/Abstract] OR ""men""[Title/Abstract]) AND (""systemic lupus erythematosus""[Title] OR ""SLE""[Title])) AND ((fha[Filter]) AND (humans[Filter]) AND (english[Filter]))","1,092",08:05:43
5,#1 AND #2,Most Recent,"English, Humans","((""sex""[Title] OR ""gender""[Title] OR ""sex""[MeSH Terms] OR (""sex""[MeSH Terms] OR ""gender identity""[MeSH Terms]) OR ""male""[Title/Abstract] OR ""men""[Title/Abstract]) AND (""systemic lupus erythematosus""[Title] OR ""SLE""[Title])) AND ((humans[Filter]) AND (english[Filter]))","1,144",08:05:33
4,#1 AND #2,Most Recent,English,"((""sex""[Title] OR ""gender""[Title] OR ""sex""[MeSH Terms] OR (""sex""[MeSH Terms] OR ""gender identity""[MeSH Terms]) OR ""male""[Title/Abstract] OR ""men""[Title/Abstract]) AND (""systemic lupus erythematosus""[Title] OR ""SLE""[Title])) AND (english[Filter])","1,398",08:05:21
3,#1 AND #2,Most Recent,,"(""sex""[Title] OR ""gender""[Title] OR ""sex""[MeSH Terms] OR (""sex""[MeSH Terms] OR ""gender identity""[MeSH Terms]) OR ""male""[Title/Abstract] OR ""men""[Title/Abstract]) AND (""systemic lupus erythematosus""[Title] OR ""SLE""[Title])","1,537",08:04:51
2,systemic lupus erythematosus [Title] OR SLE[Title],Most Recent,,"""systemic lupus erythematosus""[Title] OR ""SLE""[Title]","38,892",08:04:39
1,((sex[Title] OR gender[Title]) OR (sex[MeSH Terms])) OR (gender[MeSH Terms]) OR male [Title/abstract] OR men [Title/abstract],Most Recent,,"""sex""[Title] OR ""gender""[Title] OR ""sex""[MeSH Terms] OR ""sex""[MeSH Terms] OR ""gender identity""[MeSH Terms] OR ""male""[Title/Abstract] OR ""men""[Title/Abstract]","1,804,876",08:04:04

Suppl. Table 2 Search Terms for Pubmed, last search on 29 November 2024		
#1	Search: ((sex[Title] OR gender[Title]) OR (sex[MeSH Terms])) OR (gender[MeSH Terms]) OR male [Title/abstract] OR men [Title/abstract]	1,804,876
#2	Search: systemic lupus erythematosus [Title] OR SLE[Title]	38,892
#3	#1 AND #2	1,537
Filter	English language	1,398
	Humans	1,144
	abstract available,	1,092
	Publication date 01/01/2015 to 29/11/2024	434
	#3 NOT ("case reports"[Publication Type] OR "editorial"[Publication Type])	364
	Search Terms for Cochrane reviews, last search on 03 December 2024	
	(sex OR gender OR male OR female) AND lupus in Title Abstract Keyword - (Word variations have been searched)	9

Supplementary Figure 1 Flow Chart

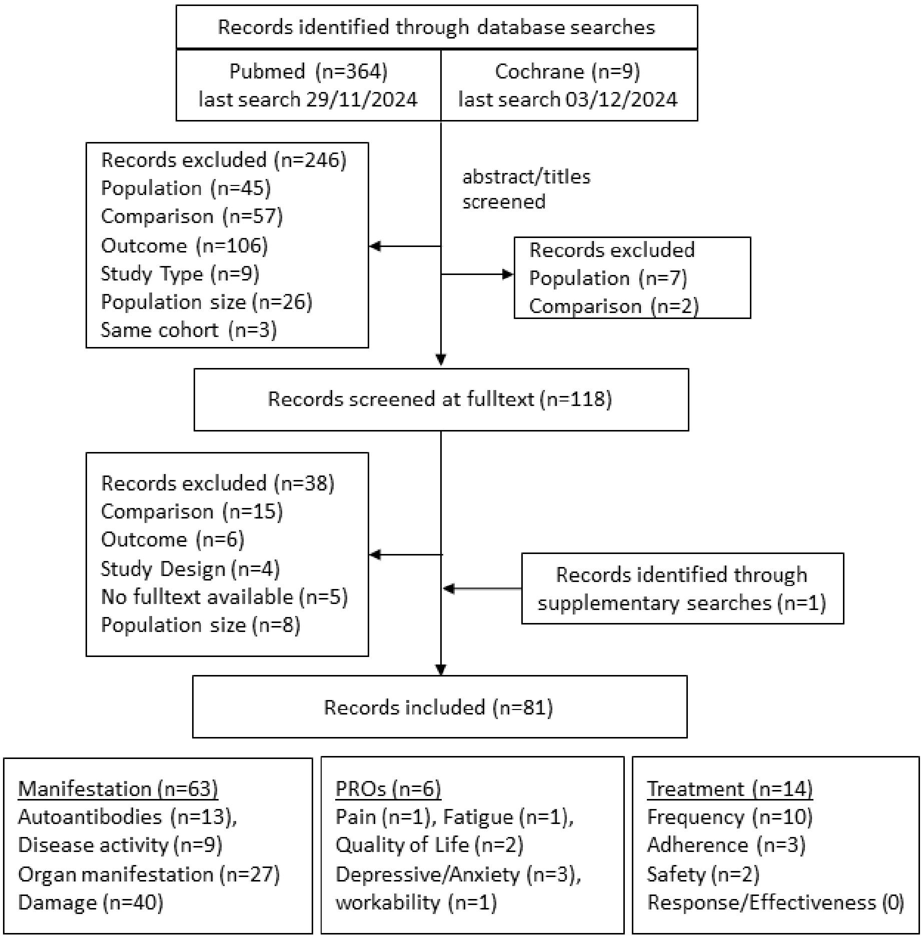

Supplementary Table S3 Characteristics of included studies

First Author	Country	Year	Study design	Study name	Study Period	Ethnicity	Total Number	Number of patients included		Ratio Women:Men	Age at onset, mean±SD (years)		Time to diagnosis, mean±SD	
								Women	Men		Women	Men	Women	Men
Adwan[1]	Jordan	2024	cohort		2018-2020	Arab	275	244	31	8:1	26±10	29±12	13.3 ± 41.8 m	4.3 ± 15.4 m
Mahmood[2]	USA	2024	MA		until 02 2021		7845	6635	1210	6:1				
Gong[3]	China	2023	cohort		2012-2021		526	476	50	10:1				
Mihailovic[4]	Switzerland	2023	cohort	SSCS	2007-2017	Caucasian	622	529	93	6:1	33 (29;57)	44 (24;46)	median0.3 y	Median 0.3 y
Rice[5]	USA	2023	cohort		2018-2022	Various	1462	1336	126	11:1				
Trentin[6]	Italy	2023	cohort		n.a.	Caucasian	417	366	51	7:1	28±11	35±14	2.2±4.1y	2.8±5.2y
Gui[7]	China	2022	cohort	CSTAR	2009-2021	Asian	8713	7918	795	10:1	35±14	37±16		
Lee[8]	Singapore	2022	MA		until 01 2020	Asian	2255	1923	332	6:1				
Ramirez[9]	Sweden	2019	cohort		n.a.	Caucasian	1226	1060	166	6:1	36±15	40±19		
Jolly[10]	US, Europe	2019	cohort	SOUL		mixed	1803	1681	122	14:1				
Shaharir[11]	Malaysia	2019	cohort		2016-2017	Asian	418	359	59	6:1	31±13	28±13		
El Hadidi[12]	Egypt	2018	cohort		1980-2016		1109	995	114	9:1	26±11	25±11		
Santamaria[13]	Colombia	2018	cohort		2012-2017		200	169	31	6:1				
Gergianaki[14]	Greece	2017	cohort		2013	Greek	750	697	53	13:1				
Riveros F.[15]	Spain	2017	cohort	RELESSER	2011-2012	Caucasian	3651	3298	353	9:1	33±14	37±17	31.7±54.5m	24.9±41.6m
Budhoo[16]	South Africa	2017	cohort			Indians African Blacks	408	372	36	10:1	29±13	33±14		
Rastin[17]	Iran	2017	cohort				98	78	20	4:1				
Munoz G.[18]	Colombia	2016	cohort			Mestizo	160	120	40				Median 4m	Median 3m
Boodhoo[19]	China	2016	MA		until 01 2016	mixed	11934	10331	1603	9:1				
Rees[20]	UK	2016	cohort	CPRD	1999-2012	NR	7732	6634	1098	6:1				

MA meta-analysis, m months, NR not reported, SD standard deviation, y years

Supplementary table S4: Presence of autoantibodies (%) in men and women with SLE

	range in studies (%)<br>men	 women	Adwan[1]	Gong[3]	Mihailovic[4]	Trentin[6]	Gui[7]	Ramirez[9]	Shaharir[11]	El Hadidi[12]	Budhoo[16]	Rastin[17]	Riveros[15]	Munoz[18]	Boodhoo[19]
N male			31	50	93	51	795	166	59	114	36	20	353	40	1603
N female			244	476	529	366	7918	1060	359	995	372	78	3298	120	10331
ANA	95-100	96-100	100/96<br>1.0		95/98<br>1.0	100/100<br>1.0	97/96<br>1.0	98/98<br>1.0		99/97<br>1.0	100/97<br>1.0		99/99<br>1.0		0.8<br>0.6;1.3
Anti-dsDNA	44-94	36-79	65/36<br>1.8	64/70<br>0.9	69/64<br>1.1	71/76<br>0.9	44/47<br>0.9	66/60<br>1.1	71/77<br>0.9	84/79<br>0.8	47/45<br>1.0	67/70<br>1.0	79/73<br>1.1	75/74<br>1.0	1.2<br>1.0;1.5
Anti-Sm	11-58	14-56	28/34<br>0.8	58/56<br>1.0	16/21<br>0.8	20/14<br>1.4	42/44<br>1.0	15/14<br>1.1	32/28<br>1.1	19/23<br>0.8	53/32<br>1.7	11/15<br>0.7	20/21<br>1.0	46/43<br>1.1	1.6<br>0.9;2.6
Anti-RNP	13-66	19-62	21/21<br>1.0	66/62<br>1.1		22/26<br>0.8	33/35<br>0.9		13/22<br>0.6		67/58<br>1.2	11/19<br>0.6	23/25<br>0.9	54/44<br>1.2	
Anti-Ro/SSA	8-54	21-54	28/21<br>1.3	48/52<br>0.9		38/36<br>1.1*	40/54<br>0.7		23/45<br>0.5	8/22<br>0.4		21/48<br>0.4	28/41<br>0.7	33/54<br>0.6	
Anti-La/SSB	0-30	11-29	21/15<br>1.4	30/29<br>1.0			14/20<br>0.7		23/20<br>1.2	5/12<br>0.4		0/11<br>0.0	16/20<br>0.8	20/22<br>0.9	
Anti-Phospholipids	26-50	24-40			37/44<br>0.8	50/40<br>1.3	26/24<br>1.1			43/42<br>1.0					
Anti-Cardiolipin	27-35	16-31	31/26<br>1.2						35/31<br>1.1				27/25<br>1.1	27/16<br>1.7	1.3<br>0.8;2.0
Anti-B2 Glycoprotein													13/14<br>0.9		
Lupus-Anticoagulant	28-39	14-30	39/30<br>1.3			35/24<br>1.5			28/14<br>2.0				34/23<br>1.5	33/23<br>1.4	2.0<br>1.5;2.6

Reported are the proportion in men (first value), the proportion in women (second value) in percent and the Odds ratio (OR: percentage men/percentage women). OR >1.4 are considered as relevantly more frequent in men while OR <0.6 are considered relevantly more frequent in women. If available, 95% confidence intervals are also reported. * in the Italian cohort, Anti-Ro/SSA or Anti-La/SSB are reported.

Supplementary table S5: Mean or median values of disease activity markers in men and women with SLE

First Author	Adwan[1]	Mihailovic[4]	Gui[7]	Jolly[10]	Santamaria[13]	Riveros[15]	Munoz[18]	Boodhoo[19]
N male	31	93	795	122	31	353	40	1603
N female	244	529	7918	1681	169	3298	120	10331
SLEDAI		Median (IQR)<br>4 (2-9)/4 (2-8)	Mean (SD)<br>8.3 (7.2) /8.3 (7.5)	Median (IQR)<br>2.0 (4.0)/2.0 (4.0)	Mean (SD)<br>17.4(10)/14(10)<br>% >10:<br>OR 2.7 (1,1; 6.3)	% ≥6<br>15/14<br>OR 1.1	Median (IQR)<br>15 (12-22)/12 (8-21)	
Low C3, %	68%/49%<br>OR 1.4				77(37)/80(43)<br>NS			OR 1.4 (1.1;1.8)
Low C4, %	55%/42%<br>OR 1.3				17(10)/16(12)<br>NS<br><10 mg/dl: OR 0.4			OR 0.98 (0.7;1.3)
ECLAM					mean (SD): 5.5 (2.4)/4.0 (2.2)<br>>5: OR 2.7 (1.2; 5.8)			
ESR		mean<br>16 (7;35)/14 (7;33)			Mean<br>55(36)/49(40)			
Physician global		active: 56%/54%<br>1.0		1.0/0.4<br>median				

Reported are mean (standard deviation), median (inter quartil range) or the percentage in men (always the first value) and women (second value) and the Odds ratio (OR: percentage men/percentage women). OR >1.4 are considered as relevantly more frequent in men while OR <0.6 are considered relevantly more frequent in women. If available, 95% confidence intervals are also reported.

Supplementary table S6: Presence of clinical manifestations (%) in men and women

First Author	Adwan[1]	Mihailovic[4]	Rice[5]	Trentin[6]	Gui[7]	Lee[8]	Ramirez[9]	Shaharir[11]	El Hadidi[12]	Budhoo[16]	Santamaria[13]	Gergianaki[14]	Rastin[17]	Riveros[15]	Munoz[18]	Boodhoo[19]
Number of patients	275	622	1462	417	8713	2255	1226	418	1109	408	200		98	3651	160	11934
Constitutional				27/20<br>1.4												
Fever	48/37<br>1.3				18/15<br>1.2				62/40<br>1.6							
Mucocutaneous				56/65<br>0.9	36/40<br>0.9			31/43<br>0.7							75/83<br>0.9	
Malar rash	45/52<br>0.9	30/39<br>0.8		Skin 35/40<br>0.9		1.0<br>0.7;1.0	40/56<br>0.7		42/49<br>0.9	41/50<br>0.8				44/56<br>0.8	23/36<br>0.6	0.7<br>0.5;0.9
Discoid rash	9.7/4.5<br>2.2	15/21<br>0.7				1.2<br>0.5;2.6	19/24<br>0.8		6.1/5.3<br>1.2	47/26<br>1.8				24/21<br>1.1	10/14<br>0.7	1.2<br>0.8;1.7
Photosensitivity	55/62<br>0.9	28/51<br>0.5				0.7<br>0.5;1.0	43/67<br>0.6		44/46<br>1.0	58/68<br>0.9				46/62<br>0.7	33/44<br>0.8	0.7<br>0.6;0.8
Oral ulcers	32/41<br>0.8	23/30<br>0.8				1.1<br>0.7;1.8	16/25<br>0.6		34/35<br>1.0	53/50<br>1.1			16/1.3<br>12.3	35/47<br>0.7	35/30<br>1.2	0.7<br>0.6;0.8
Alopecia	19/55<br>0.3					0.3<br>0.2;0.5			28/42<br>0.7					16/38<br>0.4	18/53<br>0.3	0.4<br>0.3;0.5
Raynaud	36/56<br>0.6		*/18						10/15<br>0.7					24/35<br>0.7	13/15<br>0.9	0.8<br>0.5;1.2
Muskuloskeletal								46/63<br>0.7								
Arthritis	48/45<br>1.1	57/74<br>0.8		71/49<br>1.4	22/30<br>0.7	0.7<br>0.6;0.98	70/79<br>0.9		67/78<br>0.9	81/81<br>1.0			47/61<br>0.8	74/78<br>0.9	60/62<br>1.0	0.7<br>1.3;1.8
Haematological	61/62<br>1.0	64/60<br>1.1		46/67<br>0.7		1.2<br>0.8;1.7	60/62<br>1.0	68/63<br>1.1		83/74<br>1.1	74/68<br>1.1	47/28<br>1.7	32/44<br>0.7		80/88<br>0.9	0.9<br>0.7;1.2
Thrombozytopenia	32/30<br>1.1				21/19<br>1.1	1.6<br>1.0;2.5	16/18<br>0.9		17/13<br>1.3	36/25<br>1.4				4.7/6.3<br>0.7	10/22<br>0.5	1.3<br>1.1;1.6
Leukopenia	42/39<br>1.1				21/25<br>0.8	0.7<br>0.4;1.1	40/43<br>0.9		49/45<br>1.1	58/42<br>1.4				52/62<br>0.8		0.8<br>0.6;1.0
Venous TE	29/20<br>1.5								12/4<br>3.0					15/9<br>1.7	5.0/4.2<br>1.2	
Haemolytic anaemia						1.1<br>0.7;1.7			7.9/10.4<br>0.8	22/22<br>1.0				7.7/9<br>0.9	18/13<br>1.4	1.0<br>0.8;1.3
APS	32/14<br>2.3		19/13<br>1.5	28/16<br>1.8				8.5/8.4<br>1.0	15/11<br>1.4		19/18<br>1.1			17/14<br>1.2	2.5/5.8<br>0.4	
Serositis	32/26<br>1.2	34/27<br>1.3		16/7<br>2.3	15/12<br>1.3	1.2<br>0.9;1.7	56/41<br>1.4	10/12<br>0.8		25/19<br>1.3		28/14<br>2.0				1.5<br>1.3;1.8

Pleuritis		28/20<br>1.4					47/36<br>1.3		28/29<br>1.0					35/21<br>1.7	35/25<br>1.4	1.3<br>1.1;1.5
Pericarditis		17/18<br>0.9					27/17<br>1.6		13/8<br>1.6		36/27<br>1.3			23/15<br>1.5	18/14<br>1.3	1.2<br>0.97;1.5
Nephritis	58/36<br>1.6	42/37<br>1.1	25/18<br>1.4	43/45<br>1.0	44/32<br>1.4	1.9<br>1.3;2.8	54/30<br>1.8	78/64<br>1.2	44/32<br>1.4	42/39<br>1.1	61/52<br>1.2	26/12<br>2.2	68/37<br>1.8		55/51<br>1.1	1.5<br>1.3;1.8
Neuropsychiatric	16/11<br>1.5	8/9<br>0.9		14/10<br>1.4	6.5/6.2<br>1.0	1.1<br>0.5;2.3	11/9<br>1.2	19/13<br>1.5	3.5/2.4<br>1.5			13/3<br>4.3	11/22<br>0.5			1.2<br>0.8;1.7
Seizures		6/4<br>1.5					10/7<br>1.4		3.5/4<br>0.9	16.7/9.1<br>1.8				11/6.4<br>1.7	2.5/5.0<br>0.5	1.2<br>0.9;1.5
Psychosis		1/6<br>0.2					3.1/1.7<br>1.8		3.5/2.4<br>1.5	5.6/8.6<br>0.7				2.6/2.3<br>1.1	5.0/6.7<br>0.7	0.8<br>0.5;1.1

Reported are the proportion in men (first value), the proportion in women (second value) in percent and the Odds ratio (OR: percentage men/percentage women). OR >1.4 are considered as relevantly more frequent in men while OR <0.6 are considered relevantly more frequent in women. OR between 0.8 and 1.3 are considered as no or little difference. If available, 95% confidence intervals are also reported. APS: Antiphospholipid Syndrome, TE: Thromboembolism. The study from Shaharir is also included in the meta-analysis from Lee et al.

Supplementary Table S7: Mean or median values or presence of damage in men and women

First Author	Arkema[21]	Mihailovic[4]	Rice[5]	Trentin[6]	Gui[7]	Jolly[10]	Shaharir[11]	Gergianaki[14]	Tarr[22]	Riveros[15]	Yu[23]	Rees[20]
Number of patients	126	622	1462	417	8713	1803	418		357	3651	1196	1098
SDI	mean<br>0.48/0.18	mean<br>1(0.3)/0(0.2)		median<br>1/1	>1: 26/22<br>1.2	median<br>1.0/0.0		≥1: 28/18 y1<br>1.6	Mean<br>2.0/1.9			
Ocular					1.8/1.4<br>1.3		1.7/5.0<br>0.3					
Neuropsychiatric					4.4/3.2<br>1.4							
Renal		12/11<br>1.1	*/5%<br>n.a.		11/7<br>1.6		25/9<br>2.8					M 1.2 (0.6;2.4)*<br>F 0.8 (0.6;1.1), IRR 1.5
Pulmonary					5/4<br>1.3		7.3/4.2<br>1.7					
Cardiovascular		24/12<br>2.0	46/33<br>1.4		3.1/1.7<br>1.8		20/7<br>2.9					M 8.3 (6.3;11.0)<br>F 4.7 (4.1;5.4), IRR 1.8
Hypertension		15/4<br>3.8		34/27<br>1.3						41/28<br>1.5	44/27<br>1.6	
Dyslipidemia		29/21<br>1.4		17/16<br>1.1						39/31<br>1.3	22/13<br>1.7	
Peripheral vascular		2/3<br>0.7			2.4/0.8<br>3.0							
Gastrointestinal					1.5/1.0<br>1.5							
Muskuloskeletal					4.7/3.5<br>1.3							
Osteoporosis				17/24<br>0.7								M 6.2 (4.6–8.5)<br>F 12.3 (11.2;13.5), IRR 0.5
Skin					4.0/4.2<br>1.0							
Endocrine (Diabetes)		8/4<br>2.0		0/3.6<br>0.0	1.1/0.5<br>2.2		7.3/8.1<br>0.9			5.7/4.9<br>1.2	12/6.3<br>1.9	
Sjögren				11/6.6<br>1.7								
Gonadal					0.1/0.1<br>1							
Malignancies					0.1/0.2<br>0.5		1.5/1.7<br>0.9			7/5.6<br>1.3		M 19.1 (15.9;23.1)<br>F 14.1 (12.9;15.4), IIR 1.4
Infections												M 151 (130;175)<br>F 189 (178;201), IRR 0.8

Reported are mean or median values or the proportion in men (first value), the proportion in women (second value) in percent and the Odds ratio (OR: percentage men/percentage women). OR >1.4 are considered as relevantly more frequent in men while OR <0.6 are considered relevantly more frequent in women. OR between 0.8 and 1.3 are considered as no or little difference. If available, 95% confidence intervals are also reported. Rees et al reported Incidence Rates (IR) with 95% CI per 1,000, IRR: Incidence Rate Ratio, y year

Supplementary Table S8: Patient-reported outcomes

First Author		Rice[5]	Blomjous[24]	Jolly[10]	Macedo[25]
N female		1336	SR	1681	54
N male		126		122	54
Pain, moderate to severe		59/70 (OR 0.8)			
Health perception, fair to poor		57/58 (OR 1.0)			
Fatigue, moderate to severe		55/68 (OR 0.8)			
Pain, moderate to severe		59/71 (OR 0.8)			
Lack of confidence in completing medical forms		23/12 (OR 1.9)			
Requiring help reading health-related materials		30/19 (OR 1.6)			
Having difficulty understanding written health information		23/18 (OR 1.3)			
Risk of work loss			F OR 1.9 (1.2;3.0)		
self-reported inability to work			OR 4.5 (1.3;15.8)		
Lupus-PRO HRQoL (median)	Lupus symptoms			83/75	
	Cognition			75/63	
	Lupus Medications			63/75	
	Procreation			100/100	
	Physical Health			90/85	
	Pain/Vitality			75/65	
	Emotional Health			58/58	
	Body Image			80/80	
Lupus-PRO nonHRQoL	Desires-Goals			68/69	
	Social Support			63/75	
	Coping			58/67	
	Satisfaction with Treatment			75/75	
Depressive symptoms (BDI)					22/39 (OR 0.6)
Anxiety					17/39 (OR 0.4)

Reported are the proportion in men (first value), the proportion in women (second value) in percent and the Odds ratio (OR: percentage men/percentage women). OR >1.4 are considered as relevantly more frequent in men while OR <0.6 are considered relevantly more frequent in women. SR systematic review

Supplementary Table 9: Treatment

First Author	Lao[26]	Mihailovic[4]	Rice[5]	Trentin[6]	Gui[7]	Santamaria[13]	Riveros[15]
N male	346	93	126	51	795	31	353
N female	2285	529	1336	366	7918	169	3298
	≥1 year follow up	Study baseline	ever	Ever/at last visit	start	Past 3 months	ever
Glucocorticoids	67/68 (OR 1.0)	76/63 (OR 1.2)		94/98 (OR 1.0)	88/86 (OR 1.0)	94/97 (OR 1.0)	92/89 (OR 1.0)
Antimalarials	aOR 0.5 (0.4;0.7)	73/79 (OR 0.9)			69/72 (OR 1.0)	32/46 (OR 0.7)	77/84 (OR 0.9)
Methotrexate	19/26 (OR 0.7)		23/24 (OR 1.0)	16/21 (OR 0.8)		13/15 (OR 0.9)	14/17 (OR 0.8)
Mycophenolate	30/27 (OR 1.1)		32/25 (OR1.3)	42/33 (OR 1.3	12/14 (OR0.9)	16/10 (OR 1.6)	18/15 (OR 1.2)
Ciclosporin A				14/26 (OR 0.5)			
Cyclophposphamide			-*/3	32/36 (OR 0.9)	23/16 (OR 1.4)	23/8 (OR 2.9)	32/21(OR 1.5)
Azathioprine	35/40 (OR 0.9)		-*/19	18/39 (OR 0.5)		23/20 (OR 1.2)	32/33 (OR 1.0)
Rituximab			-*/6	14/13 (OR 1.1)			6.9/6.5 (OR 1.1)
Belimumab			-*/8	4/12 (OR 0.3)			

Reported are the proportion in men (first value), the proportion in women (second value) in percent and the Odds ratio (OR: percentage men/percentage women). OR >1.4 are considered as relevantly more frequent in men while OR <0.6 are considered relevantly more frequent in women. *In the US cohort, values corresponding to less than 20 men have not been reported. aOR adjusted Odds ratio

References

1. Adwan MH, Qasem U, Atawnah SY, et al. Insights into systemic lupus erythematosus: a retrospective observational study of clinical features, autoantibodies, and gender-related differences. *Rheumatol Int* 2024;44(7):1255-63. doi: 10.1007/s00296-024-05592-7 [published Online First: 20240508]
2. Mahmood SB, Aziz M, Malepati D, et al. Evaluating Sex Differences in the Characteristics and Outcomes of Lupus Nephritis: A Systematic Review and Meta-Analysis. *Glomerular Dis* 2024;4(1):19-32. doi: 10.1159/000535981 [published Online First: 20240104]
3. Gong M, Dai L, Xie Z, et al. Serological and clinical associations of autoantibodies in Chinese patients with new-onset systemic lupus erythematosus. *Sci Rep* 2023;13(1):10101. doi: 10.1038/s41598-023-37100-5 [published Online First: 20230621]

4. Mihailovic J, Ribi C, Chizzolini C, et al. Worse cardiovascular and renal outcome in male SLE patients. *Sci Rep* 2023;13(1):18628. doi: 10.1038/s41598-023-45171-7 [published Online First: 20231030]
5. Rice C, Ayyala DN, Shi H, et al. Sex and Racial Differences in Systemic Lupus Erythematosus Among US Adults in the All of Us Research Program. *Arthritis Care Res (Hoboken)* 2023;75(10):2096-106. doi: 10.1002/acr.25093 [published Online First: 20230412]
6. Trentin F, Signorini V, Manca ML, et al. Gender differences in SLE: report from a cohort of 417 Caucasian patients. *Lupus Sci Med* 2023;10(1) doi: 10.1136/lupus-2022-000880
7. Gui Y, Bai W, Xu J, et al. Sex differences in systemic lupus erythematosus (SLE): an inception cohort of the Chinese SLE Treatment and Research Group (CSTAR) registry XVII. *Chin Med J (Engl)* 2022;135(18):2191-99. doi: 10.1097/cm9.0000000000002360 [published Online First: 20220920]
8. Lee MH, Koh JWH, Ng CH, et al. A meta-analysis of clinical manifestations in asian systemic lupus erythematous: The effects of ancestry, ethnicity and gender. *Semin Arthritis Rheum* 2022;52:151932. doi: 10.1016/j.semarthrit.2021.12.004 [published Online First: 20211230]
9. Ramírez Sepúlveda JI, Bolin K, Mofors J, et al. Sex differences in clinical presentation of systemic lupus erythematosus. *Biol Sex Differ* 2019;10(1):60. doi: 10.1186/s13293-019-0274-2 [published Online First: 20191216]
10. Jolly M, Sequeira W, Block JA, et al. Sex Differences in Quality of Life in Patients With Systemic Lupus Erythematosus. *Arthritis Care Res (Hoboken)* 2019;71(12):1647-52. doi: 10.1002/acr.23588 [published Online First: 20181220]
11. Shaharir SS, Kadir WDA, Nordin F, et al. Systemic lupus erythematosus among male patients in Malaysia: how are we different from other geographical regions? *Lupus* 2019;28(1):137-44. doi: 10.1177/0961203318812676 [published Online First: 20181120]
12. El Hadidi KT, Medhat BM, Abdel Baki NM, et al. Characteristics of systemic lupus erythematosus in a sample of the Egyptian population: a retrospective cohort of 1109 patients from a single center. *Lupus* 2018;27(6):1030-38. doi: 10.1177/0961203317751856 [published Online First: 20180210]
13. Santamaría-Alza Y, Motta JZN, Fajardo-Rivero JE, et al. Systemic lupus erythematosus, gender differences in Colombian patients. *Clin Rheumatol* 2018;37(9):2423-28. doi: 10.1007/s10067-018-4161-8 [published Online First: 20180602]
14. Gergianaki I, Fanouriakis A, Repa A, et al. Epidemiology and burden of systemic lupus erythematosus in a Southern European population: data from the community-based lupus registry of Crete, Greece. *Ann Rheum Dis* 2017;76(12):1992-2000. doi: 10.1136/annrheumdis-2017-211206 [published Online First: 20170805]
15. Riveros Frutos A, Casas I, Rúa-Figueroa I, et al. Systemic lupus erythematosus in Spanish males: a study of the Spanish Rheumatology Society Lupus Registry (RELESSER) cohort. *Lupus* 2017;26(7):698-706. doi: 10.1177/0961203316673728 [published Online First: 20161031]
16. Budhoo A, Mody GM, Dubula T, et al. Comparison of ethnicity, gender, age of onset and outcome in South Africans with systemic lupus erythematosus. *Lupus* 2017;26(4):438-46. doi: 10.1177/0961203316676380 [published Online First: 20161112]
17. Rastin M, Mahmoudi M, Sahebari M, et al. Clinical & immunological characteristics in systemic lupus erythematosus patients. *Indian J Med Res* 2017;146(2):224-29. doi: 10.4103/ijmr.IJMR_1356_15
18. Muñoz-Grajales C, González LA, Alarcón GS, et al. Gender differences in disease activity and clinical features in newly diagnosed systemic lupus erythematosus patients. *Lupus* 2016;25(11):1217-23. doi: 10.1177/0961203316635286 [published Online First: 20160225]
19. Boodhoo KD, Liu S, Zuo X. Impact of sex disparities on the clinical manifestations in patients with systemic lupus erythematosus: A systematic review and meta-analysis. *Medicine (Baltimore)* 2016;95(29):e4272. doi: 10.1097/md.0000000000004272

20. Rees F, Doherty M, Grainge M, et al. Burden of Comorbidity in Systemic Lupus Erythematosus in the UK, 1999-2012. *Arthritis Care Res (Hoboken)* 2016;68(6):819-27. doi: 10.1002/acr.22751
21. Arkema EV, Saleh M, Simard JF, et al. Epidemiology and Damage Accrual of Systemic Lupus Erythematosus in Central Sweden: A Single-Center Population-Based Cohort Study Over 14 Years From Ostergotland County. *ACR Open Rheumatol* 2023;5(8):426-32. doi: 10.1002/acr2.11585 [published Online First: 20230719]
22. Tarr T, Papp G, Nagy N, et al. Chronic high-dose glucocorticoid therapy triggers the development of chronic organ damage and worsens disease outcome in systemic lupus erythematosus. *Clin Rheumatol* 2017;36(2):327-33. doi: 10.1007/s10067-016-3492-6 [published Online First: 20161126]
23. Yu KH, Kuo CF, Chou IJ, et al. Risk of end-stage renal disease in systemic lupus erythematosus patients: a nationwide population-based study. *Int J Rheum Dis* 2016;19(11):1175-82. doi: 10.1111/1756-185x.12828 [published Online First: 20160210]
24. Blomjous BS, Gajadin GRS, Voskuyl AE, et al. Work participation in patients with systematic lupus erythematosus: a systematic review. *Rheumatology (Oxford)* 2022;61(7):2740-54. doi: 10.1093/rheumatology/keab855
25. Macêdo EA, Appenzeller S, Costallat LT. Gender differences in systemic lupus erythematosus concerning anxiety, depression and quality of life. *Lupus* 2016;25(12):1315-27. doi: 10.1177/0961203316638934 [published Online First: 20160316]
26. Lao C, Dantzig PV, Tugnet N, et al. Treatment patterns in patients with systemic lupus erythematosus in New Zealand. *Lupus* 2024;33(11):1260-73. doi: 10.1177/09612033241274911 [published Online First: 20240816]
